# Editorial: The mechanism of sudden unexpected death in epilepsy and the specific forensic diagnostic indicators in sudden death with a negative autopsy

**DOI:** 10.3389/fneur.2023.1265787

**Published:** 2023-08-18

**Authors:** Beixu Li, Likun Wang, Bin Tu

**Affiliations:** ^1^School of Policing Studies, Shanghai University of Political Science and Law, Shanghai, China; ^2^Shanghai Fenglin Forensic Center, Shanghai, China; ^3^Emergency Department of Internal Medicine-Neurology, Affiliated Hospital of Guizhou Medical University, Guiyang, Guizhou, China; ^4^Comprehensive Epilepsy Center, Department of Neurology, College of Physicians and Surgeons, Columbia University, New York, NY, United States

**Keywords:** sudden unexpected death, places of confinement, sudden unexpected death in epilepsy, molecular mechanism, forensic diagnostic indicators, artificial intelligence

Research on sudden unexpected death (SUD) is essential to clinical and forensic medicine. The diagnosis of the cause of SUD is challenging and is a research hotspot in forensic medicine, especially in cases without specific diagnostic indicators and deaths from non-natural causes, including SUD in epilepsy (SUDEP). Further, the accurate diagnosis of the cause and manner of SUD is necessary for judicial purposes to ascertain facts, settle disputes, and resolve conflicts, particularly in confined places such as prisons, detention centers, and rehabilitation centers, and in cases of SUD of healthy middle-aged and young adults.

SUDEP was defined in 2012 as a category of death and is classified into seven subtypes: [1] definite SUDEP, characterized by a sudden, unexpected, witnessed or unwitnessed, non-traumatic, and non-drowning death, occurring in benign circumstances in individuals with epilepsy with or without evidence of seizures and excluding documented status epilepticus (seizure duration longer than 30 min or seizures without recovery), in which postmortem examination does not reveal a definite cause of death, [2] definite SUDEP plus, [3] probable SUDEP/probable SUDEP plus, [4] possible SUDEP, [5] near-SUDEP/near-SUDEP plus, [6] non-SUDEP, and [7] unclassified (1). SUDEP is diagnosed by excluding other causes that may have led to death. Diagnosis is controversial because of the lack of specific forensic markers and the tendency to occur more frequently among young people (2), as demonstrated in the present study. Mechanisms underlying SUDEP are incompletely understood but may be associated with seizure-related cardiac dysfunction, respiratory depression, autonomic nervous dysfunction, and brain dysfunction in the postictal phase (3).

This study focuses on the characteristics of SUDEP, underlying molecular mechanisms, and predictors of SUD with negative autopsy. The study evaluated seven original research articles and two reviews, including six studies from China, two from Canada, and one from Italy.

Retrospective and comparative studies provided autopsy data on SUDEP, elucidating its pathological characteristics. Patients who underwent SUDEP show no lethal pathological changes but rather exhibit mild neurological, respiratory, and cardiovascular abnormalities, in line with the definition of SUDEP and its association with young age and prone position. Zhang, Zhang et al. innovatively compared autopsy and toxicological findings of SUDEP and other causes of death in individuals with epilepsy. Yan et al. evaluated three cases of SUDEP from their forensic center from 2011 to 2020 and 385 reported cases of SUDEP. Moreover, these authors discussed the importance of performing comprehensive brain examinations for suspected cases of SUDEP and evaluating the safety and effectiveness of antiepileptic drugs. Argo et al. analyzed four cases of SUDEP providing valuable data for the diagnosis of SUDEP in terms of pathological characteristics, circumstance factors, and the relationship between antiepileptic drugs and SUDEP. These autopsy data elucidate the pathogenesis and causes of SUDEP.

Two review articles evaluated the mechanism and diagnostic practices of SUDEP. Sun et al. found that SUDEP was associated with inherited cardiac ion channel diseases and severe obstructive sleep apnea. Underlying mechanisms involved decreased heart rate variability (HRV) and prolonged QT interval, potentially leading to arrhythmias; laryngospasm; amygdala activation; adenosine neuromodulation; and the inhibition of 5-HT neuronal activity. Nonetheless, little is known about the molecular mechanisms of SUDEP and risk predictors. Sun and Wang discussed current forensic methods for SUDEP diagnosis, the reasons for the low rate of diagnosis of SUDEP, and the prospects of molecular autopsy in forensic pathology. The authors suggested standardizing a testing protocol for SUDEP to facilitate data sharing and research collaboration worldwide.

Zhang, Ma et al. identified miRNA-mRNA regulatory networks associated with the glutamatergic system in a rat model of post-traumatic epilepsy (PTE) by transcriptome sequencing and bioinformatics analysis. Some miRNA-mRNA interaction pairs were involved in the development of PTE and are thus potential predictors of the risk of SUDEP. Lamrani et al. measured EEG parameters and HRV to assess autonomic function and the risk of SUDEP. HRV patterns and unusual cardiorespiratory manifestations indicated autonomic abnormalities that could predict an increased risk of SUDEP. Based on clinical data prior to death in SUDEP and non-SUDEP patients, Gravitis et al. used single spectrum analysis, independent component analysis, and cross-frequency phase-phase coupling to develop a novel metric to assess non-linear interactions between two ECG rhythms and predict the risk of SUDEP. These clinical data can help predict, prevent, and diagnose SUDEP.

Artificial intelligence, deep learning, and big data technology can assist in SUDEP prediction, prevention, and diagnosis (4). Argo et al. analyzed the risk of SUDEP using information technology as SUDEP and related research are gaining increased attention. Tong et al. performed a bibliometric analysis of studies on SUDEP. *Frontiers in Neurology* ranks sixth in the number of SUDEP-related articles published in the past 20 years, and its impact factor ranks third among these six journals. The United States, Europe, and Asia are leading the research on this topic (5). In turn, African studies on this topic are limited. The study by Argo et al. proved that. The present study discusses various aspects of SUDEP research including predictors of the risk of SUDEP in forensic pathology and underlying mechanisms, the incorporation of new technologies, and the current state of global research on the subject. The study aims to advance our understanding of SUDEP mechanisms and foster the identification of specific forensic diagnostic indicators associated with this condition.

## Author contributions

BL: Funding acquisition, Validation, Writing—original draft, Writing—review and editing. LW: Validation, Writing—review and editing. BT: Validation, Writing—review and editing.

## References

[1] Nashef L So EL Ryvlin P. Tomson tnifying the definitions of sudden unexpected death in epilepsy. Epilepsia J Int League Against Epilepsy. (2012) 53:227–33. 10.1111/j.1528-1167.2011.03358.x 22191982

[2] Devinsky O Hesdorffer DC Thurman DJ Lhatoo S Richerson G. Sudden unexpected death in epilepsy: epidemiology, mechanisms, and prevention. Lancet Neuro. (2016) 15:1075–88. 10.1016/S1474-4422(16)30158-2 27571159

[3] Ryvlin P Nashef L Lhatoo SD Bateman LM Bird J Bleasel A. Incidence and mechanisms of cardiorespiratory arrests in epilepsy monitoring units (MORTEMUS): a retrospective study. Lancet Neurol. (2013) 12:966–77. 10.1016/S1474-4422(13)70214-X 24012372

[4] Patel UK Anwar A Saleem S Malik P Rasul B Patel K. Artificial intelligence as an emerging technology in the current care of neurological disorders. J Neurol. (2021) 268:1623–42. 10.1007/s00415-019-09518-3 31451912

[5] Kaur T Diwakar A Kirandeep Mirpuri P Tripathi M Chandra PS. Artificial intelligence in epilepsy. Neurol India. (2021) 69:560–6. 10.4103/0028-3886.317233 34169842

